# Age-specific vaginal microecological dysbiosis associated with HPV infection: a large-scale cross-sectional study with targeted functional sequencing validation

**DOI:** 10.3389/fcimb.2025.1722367

**Published:** 2026-01-21

**Authors:** Yue Wang, HongJing Chen, YaoJia Chen, YanFang Lu, BiNa Wei, ZhiHui Wu, HangJing Gao, LuLu Feng, Fang Xie, Qing Li, WenYu Lin, XiaoQi Sun, Hao Lin, BinHua Dong, PengMing Sun

**Affiliations:** 1 College of Clinical Medicine for Obstetrics & Gynecology and Pediatrics, Fujian Medical University, Fuzhou, Fujian, China; 2 Laboratory of Gynecologic Oncology, Fujian Maternity and Child Health Hospital College of Clinical Medicine for Obstetrics & Gynecology and Pediatrics, Fujian Medical University, Fuzhou, Fujian, China; 3 Fujian Key Laboratory of Women and Children’s Critical Diseases Research, Fuzhou, Fujian, China; 4 Fuzhou Second General Maternity and Child Health Care Hospital, Fuzhou, Fujian, China; 5 Clinical Laboratory Department, Fujian Maternity and Child Health Hospital, College of Clinical Medicine for Obstetrics & Gynaecology and Paediatrics, Fujian Medical University, Fuzhou, Fujian, China; 6 School of Medical Technology and Engineering, Fujian Health College, Fuzhou, Fujian, China; 7 Department of Gynecology, Mindong Hospital of Fujian Medical University, Fuan, Fujian, China; 8 Department of Gynecology, Women and Children’s Hospital, School of Medicine, Xiamen University, Xiamen, Fujian, China; 9 School of Population Medicine and Public Health, Chinese Academy of Medical Sciences & Peking Union Medical College, Beijing, China

**Keywords:** age stratification, functional phenotype, HPV infection, metabolic reprogramming, microbiota dynamics, vaginal microecology

## Abstract

**Purpose:**

The vaginal microecological parameters as a critical immune barrier, yet their age-dependent interaction with Human papillomavirus (HPV) infection remains poorly understood. To characterize age-dependent vaginal microbiota composition and function across the female lifespan, and to evaluate the selective impact of HPV infection on microecological stability and infectious risk.

**Methods:**

A total of 23,672 women were stratified into four age groups (18–34, 35–44, 45–55, and >55 years). Vaginal microecology was evaluated using Gram-staining, pH, hydrogen peroxide (H_2_O_2_), leukocyte esterase, and sialidase assays. HPV genotyping was performed in 2,116 women. Statistical analysis employed univariate screening, LASSO regression for variable selection, and multivariate logistic regression to identify independent microecological risk factors, with Benjamini-Hochberg false discovery rate (FDR) correction applied across all tests. A targeted subset (n = 88) underwent 16S rRNA sequencing with differential taxa analysis using LEfSe and Random Forest, as well as BugBase phenotype prediction and COG/KEGG pathway analysis, to validate age-specific HPV-microbiome interactions.

**Results:**

Normal flora prevalence declined linearly with age (78.7% vs. 48.8% postmenopause, q<0.001), while microbial diversity peaked during perimenopause. HPV infection was selectively associated with increased bacterial vaginosis (BV) (41.58% vs. 36.46%, q=0.032) and sialidase activity (28.14% vs. 21.69%, q=0.002), but decreased vulvovaginal candidiasis (VVC, 10.57% vs. 15.66%, q=0.003). Functional analyses revealed HPV-driven anaerobic enrichment (*Gardnerella, Atopobium*) and profound metabolic reprogramming specifically in women aged 35–44 years, marked by upregulation of lipopolysaccharide biosynthesis (fold change [FC] = 37.3, q = 0.028), arachidonic acid metabolism (FC = 33.3, q = 0.038), and NOD-like receptor signaling (FC = 62.1, q < 0.001), with concurrent apoptosis suppression (FC = 0.35, q = 0.046). Age-stratified risk modeling identified loss of H_2_O_2_-producing *Lactobacilli* as the strongest HPV risk factor in younger women (18–34 years, adjusted odds ratio [aOR] = 0.59), whereas BV and sialidase dominated in midlife (35–44 years, aOR = 1.64); no significant risk factors emerged postmenopause.

**Conclusion:**

HPV infection selectively remodels vaginal microbiota composition and metabolic function in an age-dependent manner, with midlife women (35–44 years) representing a critical window for microbiota-based HPV prevention strategies.

## 1 Introduction

The vaginal microecosystem constitutes a key immune barrier in the female reproductive tract (Ravel et al., 2011; Qin et al., 2025), where microbiota inhibit pathogen colonization through acidification, antimicrobial secretion, and competitive exclusion (Aldunate et al., 2015; Dollard et al., 2022). This ecosystem dynamically evolves in response to hormonal changes, such as those during adolescence, pregnancy, perimenopause, and postmenopause, altering microbial structure, inflammatory tone, and susceptibility to infection (Krog et al., 2022; Shen et al., 2022). Although cross-sectional studies have suggested age-related shifts in microbial diversity, large-scale longitudinal evidence linking microecological changes to clinical infections (e.g., Bacterial vaginosis [BV] and Vulvovaginal candidiasis [VVC]) across the reproductive lifespan remains limited.

Persistent human papillomavirus (HPV) infection is the central cause of cervical cancer (Rahangdale et al., 2022), and its precancerous lesions, and their clearance or persistence depend on the regulation of the local immune-microecosystem network. *In vitro* and animal studies have demonstrated that *Lactobacilli* can suppress HPV E6/E7 oncogene expression by activating the interferon pathway and maintaining an acidic microenvironment. In contrast, BV-driven anaerobic overgrowth degrades the mucosal barrier and recruits immunosuppressive cells (e.g., regulatory T cells), thereby promoting persistent viral infection (Rosca et al., 2020). Existing large-scale studies have focused predominantly on BV, resulting in a lack of quantitative data on HPV risk for other vaginitides, including aerobic vaginitis (AV), VVC, trichomoniasis (TV), and cytolytic vaginosis (CV). While Maswanganye et al. and Liu et al (Liu et al., 2023; Maswanganye et al., 2024). demonstrated elevated BV prevalence in HPV-positive women, their designs neither compared HPV risks across all vaginitis subtypes nor included HPV-negative controls with clinically validated microecology profiles. Second, age-stratified risk models are conspicuously absent; Li et al (Fu and Zhang, 2023; Li et al., 2024). reported higher high-risk HPV (HR-HPV) prevalence in BV/AV/VVC/TV groups (P<0.05) but did not quantify age-specific risk increments, nor did they assess CV or evaluate vaginitis-HPV associations across reproductive life stages. Feng et al (Feng et al., 2023). identified VVC as protective (OR = 0.562), yet they similarly lacked an age-disaggregated analysis. Despite growing mechanistic insights, population-level studies still lack systematic analyses that integrate “age-microbiota structure-functional phenotype-infection risk”. This limits the development of precise intervention strategies in high-risk populations.

To address these issues, this study utilized a clinically established vaginal microecology testing platform that integrates morphological and enzymatic assessments (pH, hydrogen peroxide [H_2_O_2_], leukocyte esterase, and sialidase) (Cooperative Group of Infectious Disease, Chinese Society of Obstetrics and Gynocology, Chinese Medical Association 2016), to analyze microecological data from 23,672 women across four reproductive stages. A subset of 2,116 women who underwent concurrent HPV screening was examined in further detail. Key findings were validated using 16S rRNA sequencing. Our objectives were to delineate the age-dependent trajectories of vaginal microbiota and inflammatory markers; elucidate the selective effects of HPV on microbial structure and vaginitides (e.g., BV, VVC); and identify age-stratified microbial risk profiles indicative of HPV susceptibility based on microecological parameters. By mapping microecological shifts and modeling their association with HPV, we aim to identify targeted intervention strategies for HPV blockade and advance personalized cervical cancer prevention over the female lifespan.

## 2 Materials and methods

### 2.1 Study population

This study enrolled 23,672 participants from Fujian Maternity and Child Health Hospital, affiliated with Fujian Medical University, between March 2018 and June 2021. The study was reviewed and approved by the Ethics Committee of Fujian Provincial Maternity and Children’s Hospital, Affiliated Hospital of Fujian Medical University (Approval No. 2020KY148 & 2025KY209), and informed consent was obtained from all participants. The recruited patients were required to meet the following criteria: 1) aged ≥18 years and 2) no history of cervical treatment or surgery. To isolate the natural history of HPV-microbiota composition interactions without vaccine-derived confounding, participants with any history of HPV vaccination were excluded from enrollment. The exclusion criteria were as follows: 1) pregnant or lactating women (n=7430); 2) history of treatment with any systemic or local antibacterial, antiprotozoal, or antifungal agent in the 2 weeks preceding the study (n=3124); and 3) history of sexual intercourse in the previous 3 days (n=1560). Samples from the posterior fornix of the vagina were taken by clinicians with a long cotton swab and submitted within 1 hour for examination of vaginal discharge by saline wet mount, Gram stain mount microscopy, and chemistry analyses. From the initial cohort of 23,672 women who underwent vaginal microecological testing between March 2018 and June 2021, we identified 2,116 patients receiving concurrent HPV DNA testing with genotyping during the same clinical visit. This subset comprised patients meeting standard clinical indications for co-testing based on: 1) Current Chinese cervical cancer screening guidelines; 2) Presence of gynecological symptoms requiring comprehensive evaluation; 3) Physician-initiated testing based on individualized risk assessment; 4) No study-specific selection criteria were applied beyond routine clinical protocols.

### 2.2 Microbial density, microbial diversity, and dominant microbiota

Microbial density and diversity were quantified by standardized microscopic enumeration according to the Chinese Expert Consensus on Vaginal Microecology Testing (Cooperative Group of Infectious Disease, Chinese Society of Obstetrics and Gynocology, Chinese Medical Association 2016). Density was graded as: + (1–9 bacteria/field), ++ (10–99 bacteria/field), +++ (>100 bacteria/field), or ++++ (bacterial clumping). Diversity was graded as: + (1–3 morphotypes/field), ++ (4–6 types), +++ (7–9 types), or ++++ (≥10 types). The definition of the quantification standard is shown in the **Supplementary Table S1**. Normal microbial profiles were defined as moderate-to-high scores (++/+++) for both parameters, while microbial suppression (+) indicated concurrent reductions in density and diversity, suggesting ecological depletion. The pathogenic dominant microbial morphotypes were also identified by microscopy using an oil immersion lens with Gram staining to determine the dominant microbial morphotypes (**Supplementary Table S2**). The normal dominant microbial morphotypes were considered as *Lactobacillus*, as the proportion of *Lactobacillus* was ≥70%, and the proportion of other microbes was <30%. It is essential to note that microscopy assesses dominant morphotypes visible under Gram staining (e.g., *Lactobacillus rods*, *Gardnerella coccobacilli*) rather than the complete microbial community. This approach identifies the predominant bacterial forms but cannot detect low-abundance taxa or unculturable organisms. Therefore, we refer to these findings as ‘dominant microbiota’ or ‘microbial morphotypes’ to distinguish them from the comprehensive microbiome analysis conducted via 16S rRNA sequencing in our validation subset.

### 2.3 Microbiological metabolite evaluation

Samples were used to detect pH, H_2_O_2_, leucocyte esterase, and sialidase using a vaginal multi-test kit (drying Chemoenzymatic method) (Bioperfectus Technologies Co., Ltd, Jiangsu, China), following the manufacturer’s protocol. The microscopic image of a patient with normal vaginal microecology is shown in **Supplementary Figure 1a**.

### 2.4 Aerobic vaginosis

The gold standard for AV diagnosis is wet-mount microscopy (WMM). The AV score (Donders et al., 2002) combines information regarding bacterial flora, epithelial disruption, and inflammation, creating a score from 0 to 10: 0-2 (no AV), 3-4 (mild AV), 5-6 (moderate AV), or 7-10 (severe AV) (**Supplementary Figure 1b**).

### 2.5 Bacterial vaginosis

BV was diagnosed based on the Nugent (Nugent et al., 1991) score and Clinical Amsel (Amsel et al., 1983) criteria. The Nugent score is used as the gold standard and relies on estimating relative proportions of bacterial morphotypes in a Gram-stained vaginal smear to give a score between 0 and 10. A score of <4 was normal, 4–6 was intermediate, and >6 was BV. Based on Amsel’s clinical criteria, BV was defined as fulfillment of at least three of the following four criteria: gray homogenous discharge, the presence of clue cells, vaginal pH>4.5, and a positive amine test. In this study, the Nugent scoring system was used as the diagnostic criterion. Microscopic images of BV are shown in **Supplementary Figure 1c**.

### 2.6 Cytolytic vaginosis

CV diagnosis was established through a systematic exclusion protocol: (1) All samples with clinical signs of vaginitis (discharge, itching, erythema) or microscopic epithelial cell lysis underwent comprehensive laboratory screening, including fungal culture for Candida species, Gram stain for BV and AV, and wet mount for TV; (2) Only samples testing negative for these pathogens and meeting all CV criteria were classified as CV, including abundant *Lactobacillus* morphotypes (>70% dominance), evidence of epithelial cell cytolysis, pH < 3.8, and cyclical symptom patterns (Kraut et al., 2023) (**Supplementary Figure 1d**).

### 2.7 Trichomonal vaginosis

Direct observation of vaginal secretion samples from the posterior vaginal fornix was examined by wet mount microscopy using normal saline. The wet preparations were assessed within 10 minutes of collection, as trichomonads rapidly lose motility and become more challenging to identify (Edwards et al., 2016)(**Supplementary Figure 1e**).

### 2.8 Vulvovaginal candidiasis

The diagnosis was based on a combination of clinical signs and microscopic findings, specifically the identification of spores or pseudohyphae on Gram-stained vaginal discharge smears (Sherrard et al., 2018) (**Supplementary Figure 1f**).

### 2.9 PCR-reverse dot blot HPV genotyping

Cervical exfoliated cell collection, storage, and the PCR-reverse dot blot (RDB) HPV test (Yaneng Biotech) were performed as previously study (Sun et al., 2017). HPV genotyping was performed by hybridization and RDB using strips fixed with 23 different type-specific probes, including 18 HR-HPV types (16, 18, 31, 33, 35, 39, 45, 51, 52, 53, 56, 58, 59, 66, 68, 73, 82, and 83) and 5 low-risk human papillomavirus (LR-HPV) types (6, 11, 42, 43, and 81).

### 2.10 16S rRNA gene sequencing and analysis

#### 2.10.1 Study population and sample selection for sequencing

A subset of 88 women was recruited from the cohort for deep molecular profiling using 16S rRNA gene sequencing to validate the age-dependent findings from our large-scale epidemiological analysis. Participants were stratified by age group ([A] 18–34 years; [B] 35–44 years; [C] 45–55 years; [D] >55 years) and HPV status (Negative [N] or Positive [P]), forming eight distinct groups to enable the analysis of age-gated HPV-associated microecological dysbiosis.

Sample size justification: To address concerns about statistical power, we conducted α-diversity power analyses (Shannon index) stratified by age group. The results demonstrate that the sample size (n = 11/group) is adequate for validation in the focal age stratum (35–44 years, HPV+ vs. HPV-), with Cohen’s d = 1.62, power> 95%, α = 0.05, exceeding the 80% threshold for microbiome studies (Ferdous et al., 2022). *Post-hoc* analysis revealed sufficient power for α-diversity comparisons. Still, it acknowledged limitations for low-prevalence taxa (<5% abundance), which were therefore interpreted with caution, using Random Forest importance metrics that are robust to small sample sizes.

#### 2.10.2 DNA extraction and quantification

Genomic DNA was extracted from vaginal swab samples using the E.Z.N.A.^®^ Mag-Bind^®^ Soil DNA Kit (Omega Bio-Tek) according to the manufacturer’s protocol. Mechanical lysis was performed via bead-beating for 5 minutes at 30 Hz, and enzymatic lysis with proteinase K at 55°C for 30 minutes to ensure efficient disruption of Gram-positive bacterial cell walls. DNA was purified using magnetic bead binding, eluted in 50 μL TE buffer, and quantified using the Qubit 3.0 DNA Kit (Invitrogen). All extracted DNA samples were stored at -80°C until further processing.

#### 2.10.3 PCR amplification and library preparation

The V3-V4 hypervariable regions of the 16S rRNA gene were amplified using primers 338F (5’-ACTCCTACGGGAGGCAGCAG-3’) and 806R (5’-GGACTACHVGGGTWTCTAAT-3’) with Illumina adapter overhangs. PCR reactions (25 μL) contained 12.5 μL KAPA HiFi HotStart ReadyMix, 1 μL each primer (10 μM), 5 μL template DNA (10 ng), and 5.5 μL nuclease-free water. Thermal cycling conditions consisted of initial denaturation at 95°C (3 min), followed by 30 cycles of 95°C (30 s), 55°C (30 s), and 72°C (30 s), with final extension at 72°C (5 min). Individual PCR products were purified using AMPure XP beads (Beckman Coulter), quantified via Qubit 3.0 Fluorometric High-Sensitivity dsDNA Assay (Invitrogen), and normalized to equimolar concentrations (5 ng per sample) before pooling for library construction using the KAPA LTP Library Preparation Kit (Kapa Biosystems).

#### 2.10.4 Sequencing and raw data generation

Pooled libraries were sequenced on the Illumina MiSeq platform (Illumina, San Diego, CA) using the 2×300 bp paired-end protocol with the MiSeq Reagent Kit v3 at Sangon Biotech (Shanghai, China). Raw sequence files were generated in FASTQ format, comprising paired-end R1 and R2 files for each sample.

#### 2.10.5 Bioinformatic analysis

##### 2.10.5.1 Quality control and merging

Adapter sequences were trimmed using cutadapt v1.18. Paired-end reads were merged using PEAR v0.9.8 based on overlap relationships. Quality filtering was performed using PRINSEQ v0.20.4 with a sliding window approach (10 bp window, average quality ≥20), removing low-complexity sequences and reads containing ambiguous bases (N). Post-QC read counts per sample ranged from 38,381 to 129,869 (median: 70,167, mean: 74,190), representing 85-99.9% retention of raw reads (**Supplementary Table S3**).

##### 2.10.5.2 OTU clustering

Operational taxonomic units (OTUs) were clustered at 97% similarity using Usearch v11.0.667 with chimera removal. Representative sequences were classified using the RDP classifier v2.12 against the SILVA v138.1 database.

##### 2.10.5.3 Diversity analysis

α-diversity indices (Richness, ACE, Chao1, Shannon, Simpson) were calculated using mothur v1.43.0. β-diversity analysis was performed using weighted and unweighted UniFrac distances. Principal coordinate analysis (PCoA) was conducted using the R vegan package v2.5-6.

##### 2.10.5.4 Functional prediction

Microbial phenotypes were predicted using BugBase v0.1.0. Functional potential was inferred using PICRUSt2 against KEGG and COG (Clusters of Orthologous Groups) databases. The NSTI (Nearest Sequenced Taxon Index) scores (**Supplementary Table S4**) ranged from 0.003 to 0.116, indicating good prediction accuracy.

##### 2.10.5.5 Statistical analysis

Differential taxa were identified using Random Forest (for stability in small cohorts) and LEfSe (with an emphasis on effect size), with concordant top-ranked taxa prioritized (q < 0.05).

#### 2.10.6 Data availability

Raw 16S rRNA sequencing data were deposited in the NCBI Sequence Read Archive (SRA) under BioProject PRJNA1377879, with BioSample accession numbers SAMN53807656–SAMN53807743 (**Supplementary Table S5**). Processed data tables are available in the **Supplementary Materials** of the manuscript.

### 2.11 Statistical analysis

All data analyses were performed using SPSS 22.0 (IBM, Chicago, IL, USA) and R version 4.5.2 (2025-10-31). Categorical variables were presented as frequencies and percentages [n (%)]. Differences between groups were assessed using the Chi-square test or Fisher’s exact test, as appropriate. A two-sided P-value<0.05 was considered statistically significant (Schober et al., 2021). To account for multiple comparisons across microecological parameters, we applied Benjamini-Hochberg false discovery rate (FDR) correction to all p-values derived from chi-square tests. Adjusted p-values (q-values) were calculated using the p.adjust function in R (v4.5.2) with method = “fdr”. Statistical significance was defined as FDR-adjusted p (q-values) < 0.05 unless otherwise specified.

To identify independent risk factors of HPV infection, we conducted a three-step analytical approach, First, univariate logistic regression analysis was performed to screen for potential risk factors among vaginal microecological parameters. Second, to address multicollinearity and overfitting in our high-dimensional microecology dataset, we employed Least Absolute Shrinkage and Selection Operator (LASSO) regression with 10-fold cross-validation using the glmnet package in R. This method optimally selected the most informative variables by shrinking less important coefficients to zero. The tuning parameter lambda (λ) was determined by minimum cross-validation error, yielding an optimal λ=0.007. Third, a multivariate logistic regression analysis was performed using the LASSO selection process, which combined clinical diagnostic evaluation (**Supplementary Table S6**). A complete model inclusion method was applied to estimate adjusted Odds Ratios (aOR) and 95% Confidence Intervals (CI). Multicollinearity was assessed using the Variance Inflation Factor (VIF), with all variables showing VIF<10, indicating no severe collinearity (**Supplementary Table S7**). To verify the stability of our findings, we performed stepwise variable adjustment analysis and age-stratified subgroup analyses. A two-sided P-value<0.05 was considered statistically significant for the final multivariate model. All analysis code and processed datasets are available upon request to ensure reproducibility.

## 3 Results

### 3.1 Age-stratified trajectory of vaginal microbiota composition, homeostasis, and infectious risk across the female lifespan

**Figure 1:**
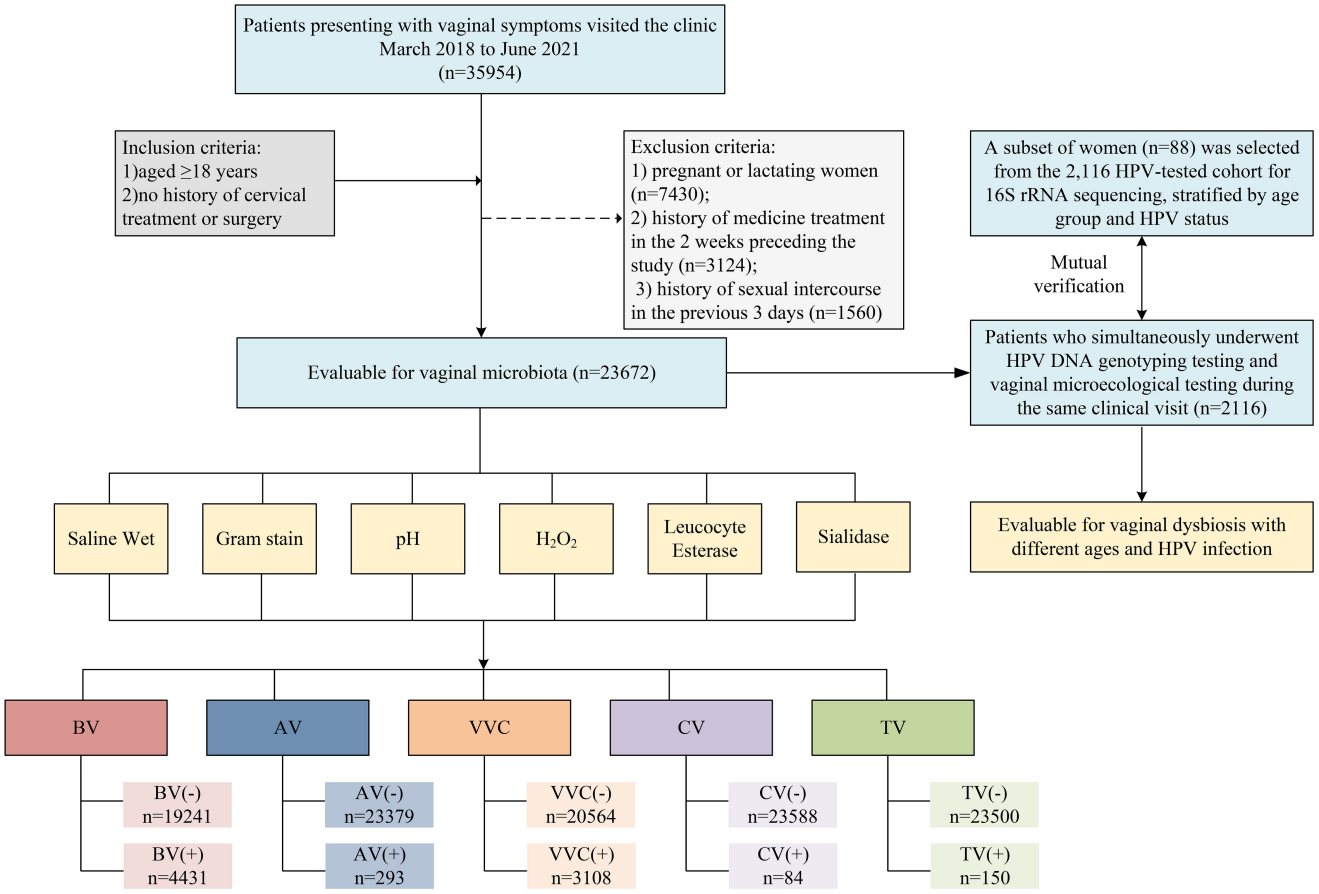
Flowchart of participants in the study. BV, bacterial vaginosis; AV, Aerobic vaginosis; CV, Cytolytic vaginosis; TV, Trichomonal vaginosis; VVC, Vulvovaginal candidiasis; HPV, human papillomavirus.

A total of 23,672 women were included (**Figure 1**) and categorized into four age-based groups (**Table 1**): Peak Reproductive Age (18–34 years, n=18,105), Advanced Reproductive Age (35–44 years, n=4,053), Perimenopausal (45–55 years, n=1,213), and Postmenopausal (>55 years, n=301). Vaginal microbiota exhibited pronounced age-dependent alterations in density, diversity, and inflammatory markers. Normal bacterial load declined linearly from 78.7% in the youngest to 48.8% in the oldest group (χ²=256.239, q<0.001). Diversity peaked in perimenopause (67.1% normal richness) before declining postmenopause (53.5%, χ²=726.681, q<0.001). Vaginal pH progressively alkalized, with pH≥4.6 increasing to 90.0% in the oldest group (χ²=134.667, q<0.001). Hydrogen peroxide-producing *Lactobacilli* reached a midlife nadir.

**Table 1:** The distribution of vaginal microbes and the expression of metabolizing enzymes in patients of different ages.

Characteristics	ALL (*n* = 23672)	Age				Statistic	*P*	FDR
		18-34(n=18105)	35-44 (n=4053)	45-55 (n=1213)	>55(n=301)			
Microbial density, *n* (%)						χ^2^ = 256.239	<0.001	<0.001
Normal	18372 (77.611)	14243 (78.669)	3185 (78.584)	797 (65.705)	147 (48.837)			
Abnormal	5300 (22.389)	3862 (21.331)	868 (21.416)	416 (34.295)	154 (51.163)			
Microbial diversity, *n* (%)						χ^2^ = 726.681	<0.001	<0.001
Normal	9734 (41.120)	6630 (36.620)	2129 (52.529)	814 (67.106)	161 (53.488)			
Abnormal	13938 (58.880)	11475 (63.380)	1924 (47.471)	399 (32.894)	140 (46.512)			
pH, n (%)						χ^2^ = 134.667	<0.001	<0.001
<4.6	6483 (27.387)	4794 (26.479)	1359 (33.531)	300 (24.732)	30 (9.967)			
≥4.6	17189 (72.613)	13311 (73.521)	2694 (66.469)	913 (75.268)	271 (90.033)			
H_2_0_2_, n (%)						χ^2^ = 186.174	<0.001	<0.001
–	1525 (6.442)	956 (5.280)	418 (10.313)	135 (11.129)	16 (5.316)			
+	22147 (93.558)	17149 (94.720)	3635 (89.687)	1078 (88.871)	285 (94.684)			
Leucocyte esterase, n (%)						χ^2^ = 1717.65	<0.001	<0.001
–	16933 (71.532)	14034 (77.514)	2388 (58.919)	442 (36.439)	69 (22.924)			
+	6739 (28.468)	4071 (22.486)	1665 (41.081)	771 (63.561)	232 (77.076)			
Sialidase, n (%)						χ^2^ = 151.012	<0.001	<0.001
–	11846 (50.042)	8670 (47.887)	2304 (56.847)	677 (55.812)	195 (64.784)			
+	11826 (49.958)	9435 (52.113)	1749 (43.153)	536 (44.188)	106 (35.216)			
BV, *n* (%)						χ^2^ = 449.470	<0.001	<0.001
–	19241 (81.282)	15170 (83.789)	3107 (76.659)	754 (62.160)	210 (69.767)			
+	4431 (18.718)	2935 (16.211)	946 (23.341)	459 (37.840)	91 (30.233)			
AV, *n* (%)						NA*	<0.001	<0.001
–	23379 (98.762)	17950 (99.144)	3994 (98.544)	1157 (95.383)	278 (92.359)			
+	293 (1.238)	155 (0.856)	59 (1.456)	56 (4.617)	23 (7.641)			
VVC, *n* (%)						χ^2^ = 45.737	<0.001	<0.001
–	20564 (86.871)	15754 (87.015)	3445 (84.999)	1071 (88.293)	294 (97.674)			
+	3108 (13.129)	2351 (12.985)	608 (15.001)	142 (11.707)	7 (2.326)			
TV, *n* (%)						NA*	<0.001	<0.001
–	23522 (99.366)	18027 (99.569)	4026 (99.334)	1172 (96.620)	297 (98.671)			
+	150 (0.634)	78 (0.431)	27 (0.666)	41 (3.380)	4 (1.329)			
CV, *n* (%)						NA*	0.031	0.031
–	23588 (99.645)	18051 (99.702)	4028 (99.383)	1209 (99.670)	300 (99.668)			
+	84 (0.355)	54 (0.298)	25 (0.617)	4 (0.330)	1 (0.332)			

*Fisher’s exact test was applied when expected cell frequencies <5 or total sample size <40. All P-values were corrected for multiple comparisons using the Benjamini-Hochberg false discovery rate (FDR) method. q<0.05 was considered statistically significant.

BV, Bacterial Vaginosis; AV, Aerobic Vaginitis; VVC, Vulvovaginal Candidiasis; TV, Trichomonas Vaginalis; CV, Cytolytic Vaginosis.

Inflammation markers rose consistently with age: leukocyte esterase positivity increased from 22.5% to 77.1% (χ²=1,717.652, q<0.001), and sialidase activity similarly elevated (χ²=151.012, q<0.001). The prevalence of BV was highest at 45–55 years (37.8%, χ²=449.470, q<0.001), whereas VVC peaked at 35–44 years (15.0%) and declined markedly to 2.3% after 55 years (χ²=45.737, q<0.001). TV was infrequent but most prevalent at 45–55 years (3.4%, q<0.001). Overall, vaginitis prevalence decreased in the oldest group (2.3%, χ²=838.962, q<0.001). Nonspecific dysbiosis showed biphasic age-related prevalence.

These findings illustrate an ecological continuum from *Lactobacilli*-dominated, low-inflammation states in youth through high-diversity midlife with increased BV risk, to an inflamed, alkaline, low-density microenvironment with reduced postmenopausal infection prevalence.

### 3.2 Associations between HPV infection and vaginal microecology

Among 2,116 women who underwent both HPV and vaginal microecological testing during the same visit (**Table 2**), HPV infection was associated with selective rather than global microecological disruption. HPV-positive women exhibited significantly higher rates of abnormal bacterial density compared to HPV-negative women (37.28% vs. 29.14%; χ²=12.651, q=0.006). Here, “abnormal density” was defined as either microbial suppression (+ score) or excessive bacterial growth (+++/++++ scores) based on our standardized microscopic criteria (see Methods 2.2). In contrast, microbial diversity remained uniformly high (>90% normal) in both groups, and neither community composition nor inflammatory tone differed significantly by HPV status (q>0.1). These data suggest HPV infection is associated with perturbations in microbial quantity and specific pathways without necessarily altering broader ecological richness or inciting overt inflammation.

**Table 2:** The vaginal microecological status in patients with different HPV infectious statuses.

Characteristics	ALL(*n* = 2116)	HPV Type		Statistic	*P*	FDR
		HPV (-)(n=1558)	HPV(+)(n=558)			
Microbial density, *n* (%)				χ²=12.275	<0.001	0.006
Normal	1454 (68.715)	1104 (70.860)	350 (62.724)			
Abnormal	662 (31.285)	454 (29.140)	208 (37.276)			
Microbial diversity, *n* (%)				χ²=0.533	0.465	0.558
Normal	1940 (91.682)	1433 (91.977)	507 (90.860)			
Abnormal	176 (8.318)	125 (8.023)	51 (9.140)			
Dominant bacteria, *n* (%)				χ²=8.877	0.064	0.122
Gram-Positive Rods	1107 (52.316)	835 (53.594)	272 (48.746)			
Gram-Negative Rods	801 (37.854)	565 (36.264)	236 (42.294)			
Gram-Positive Cocci	44 (2.079)	36 (2.311)	8 (1.434)			
Others	64 (3.025)	51 (3.273)	13 (2.330)			
Not Found	100 (4.726)	71 (4.557)	29 (5.197)			
BV, *n* (%)				χ²=4.365	0.037	0.088
–	1316 (62.193)	990 (63.543)	326 (58.423)			
+	800 (37.807)	568 (36.457)	232 (41.577)			
AV, *n* (%)				χ²=3.251	0.071	0.122
–	1999 (94.471)	1463 (93.902)	536 (96.057)			
+	117 (5.529)	95 (6.098)	22 (3.943)			
VVC, *n* (%)				χ²=8.258	0.004	0.016
–	1813 (85.681)	1314 (84.339)	499 (89.427)			
+	303 (14.319)	244 (15.661)	59 (10.573)			
TV, *n* (%)				χ²=0.607	0.436	0.558
–	2097 (99.102)	1546 (99.230)	551 (98.746)			
+	19 (0.898)	12 (0.770)	7 (1.254)			
CV, *n* (%)				χ²=0.362	0.547	0.597
–	2082 (98.393)	1535 (98.524)	547 (98.029)			
+	34 (1.607)	23 (1.476)	11 (1.971)			
pH, n (%)				χ²=1.328	0.249	0.374
<4.6	876 (41.399)	657 (42.169)	219 (39.247)			
≥4.6	1240 (58.601)	901 (57.831)	339 (60.753)			
H_2_0_2_, n (%)				χ²=4.716	0.030	0.088
–	435 (20.558)	302 (19.384)	133 (23.835)			
+	1681 (79.442)	1256 (80.616)	425 (76.165)			
Leucocyte esterase, n (%)				χ²=0.012	0.913	0.913
–	291 (13.752)	213 (13.671)	78 (13.978)			
+	1825 (86.248)	1345 (86.329)	480 (86.022)			
Sialidase, n (%)				χ²=9.157	0.002	0.015
–	1621 (76.607)	1220 (78.306)	401 (71.864)			
+	495 (23.393)	338 (21.694)	157 (28.136)			

All P-values were corrected for multiple comparisons using the Benjamini-Hochberg false discovery rate (FDR) method. q<0.05 was considered statistically significant.

BV, Bacterial Vaginosis; AV, Aerobic Vaginitis; VVC, Vulvovaginal Candidiasis; TV, Trichomonas Vaginalis; CV, Cytolytic Vaginosis.

The dominant divergence between groups was confined to specific vaginal infections. BV was more prevalent among HPV-positive women (41.58% versus 36.46%; χ²=4.365, q=0.088), and its enzymatic marker, sialidase, followed the same trend (28.14% versus 21.69%; χ²=9.157, q=0.015), suggesting an association between HPV and an anaerobe-enriched milieu. Conversely, VVC was less frequent in HPV-positive women (10.57%) than in HPV-negative women (15.66%; χ²=8.258, q=0.016), which could be consistent with either Candida-mediated immune modulation or competitive exclusion of HPV. HPV status has no significant association with AV, TV, and CV (q > 0.05).

Functional lactobacillary activity, gauged by H_2_O_2_ production, was reduced in HPV-positive women (76.17%) compared with HPV-negative controls (80.62%; χ²=4.716, q=0.088), suggesting compromised microbicidal capacity despite similar proportions of elevated pH (≥4.6) in both cohorts (>57%; χ²=1.328, q=0.374). Inflammation, as reflected by leukocyte esterase positivity, was uniformly high (>86%) and indistinguishable between the cohorts (χ²=0.012, q=0.913). The impact of HPV infection on microecology is more concentrated on the functions of the microbiota (such as *Lactobacillus* activity) and specific pathogens (such as anaerobic bacteria), rather than overall environmental instability or enhanced inflammation. These results indicate that HPV infection is a “middle-level modifier” of the microecology, altering age-related microecological trajectories through selective action.

### 3.3 Age-stratified vaginal microbiota dynamics in HPV-positive and HPV-negative women

Among 558 HPV-positive women (**Table 3**), ageing was associated with progressive dysbiosis in bacterial density and diversity, along with a shift from *Lactobacilli* (Gram-positive rods) to anaerobic dominance (Gram-negative rods). Vaginal pH increased with lactobacillary decline, although H_2_O_2_-producing *lactobacilli* paradoxically increased. BV remained highly prevalent without significant age progression, whereas VVC decreased. Inflammation was consistently elevated (>82%) in all age groups.

**Table 3:** The vaginal microecological status in HPV-positive patients of different ages.

Characteristics	ALL(*n* = 558)	HPV positive(n=558)				Statistic	*P*	FDR
		18-34yr	35-44yr	45-55yr	>55yr			
		(*n* = 251)	(*n* = 172)	(*n* = 86)	(*n* = 49)			
Microbial density, *n* (%)						χ²=9.591	0.022	0.054
Normal	350 (62.724)	171 (68.127)	107 (62.209)	49 (56.977)	23 (46.939)			
Abnormal	208 (37.276)	80 (31.873)	65 (37.791)	37 (43.023)	26 (53.061)			
Microbial diversity, *n* (%)						NA*	<0.001	0.001
Normal	507 (90.860)	240 (95.618)	164 (95.349)	74 (86.047)	29 (59.184)			
Abnormal	51 (9.140)	11 (4.382)	8 (4.651)	12 (13.953)	20 (40.816)			
Dominant bacteria, *n* (%)						NA*	<0.001	0.001
Gram-Positive Rods	272 (48.746)	140 (55.777)	88 (51.163)	32 (37.209)	12 (24.490)			
Gram-Negative Rods	236 (42.294)	99 (39.442)	80 (46.512)	42 (48.837)	15 (30.612)			
Gram-Positive Cocci	8 (1.434)	3 (1.195)	0 (0.000)	2 (2.326)	3 (6.122)			
Others	13 (2.330)	7 (2.789)	1 (0.581)	4 (4.651)	1 (2.041)			
Not Found	29 (5.197)	2 (0.797)	3 (1.744)	6 (6.977)	18 (36.735)			
BV, *n* (%)						χ²=6.207	0.102	0.136
–	326 (58.423)	152 (60.558)	94 (54.651)	45 (52.326)	35 (71.429)			
+	232 (41.577)	99 (39.442)	78 (45.349)	41 (47.674)	14 (28.571)			
AV, *n* (%)						NA*	0.095	0.136
–	536 (96.057)	243 (96.813)	168 (97.674)	79 (91.860)	46 (93.878)			
+	22 (3.943)	8 (3.187)	4 (2.326)	7 (8.140)	3 (6.122)			
VVC, *n* (%)						χ²=8.852	0.031	0.063
–	499 (89.427)	216 (86.056)	154 (89.535)	81 (94.186)	48 (97.959)			
+	59 (10.573)	35 (13.944)	18 (10.465)	5 (5.814)	1 (2.041)			
TV, *n* (%)						NA*	0.127	0.153
–	551 (98.746)	249 (99.203)	171 (99.419)	83 (96.512)	48 (97.959)			
+	7 (1.254)	2 (0.797)	1 (0.581)	3 (3.488)	1 (2.041)			
CV, *n* (%)						NA*	0.408	0.408
–	547 (98.029)	243 (96.813)	170 (98.837)	85 (98.837)	49 (100.000)			
+	11 (1.971)	8 (3.187)	2 (1.163)	1 (1.163)	0 (0.000)			
pH, n (%)						χ²=30.237	<0.001	0.000
<4.6	219 (39.247)	120 (47.809)	72 (41.860)	20 (23.256)	7 (14.286)			
≥4.6	339 (60.753)	131 (52.191)	100 (58.140)	66 (76.744)	42 (85.714)			
H_2_0_2_, n (%)						χ²=18.187	<0.001	0.001
–	133 (23.835)	72 (28.685)	46 (26.744)	13 (15.116)	2 (4.082)			
+	425 (76.165)	179 (71.315)	126 (73.256)	73 (84.884)	47 (95.918)			
Leucocyte esterase, n (%)						χ²=7.275	0.064	0.109
–	78 (13.978)	45 (17.928)	22 (12.791)	7 (8.140)	4 (8.163)			
+	480 (86.022)	206 (82.072)	150 (87.209)	79 (91.860)	45 (91.837)			
Sialidase, n (%)						χ²=4.159	0.245	0.267
–	401 (71.864)	188 (74.900)	118 (68.605)	57 (66.279)	38 (77.551)			
+	157 (28.136)	63 (25.100)	54 (31.395)	29 (33.721)	11 (22.449)			

*Fisher’s exact test was applied when expected cell frequencies <5 or total sample size <40. All P-values were corrected for multiple comparisons using the Benjamini-Hochberg false discovery rate (FDR) method. q<0.05 was considered statistically significant.

BV, Bacterial Vaginosis; AV, Aerobic Vaginitis; VVC, Vulvovaginal Candidiasis; TV, Trichomonas Vaginalis; CV, Cytolytic Vaginosis.

In 1,558 HPV-negative women (**Table 4**), *Lactobacilli* declined sharply after age 44, anaerobes peaked at 50.4% in perimenopause, and pH≥4.6 reached 90.8% in postmenopause. H_2_O_2_ positivity rebounded to 94.7%, suggesting compensatory activity. BV increased significantly with age, whereas VVC and TV decreased. AV emerged in older women.

**Table 4:** The vaginal microecological status in HPV-negative patients of different ages.

Characteristics	ALL(*n* = 1558)	HPV negative(n=1558)				Statistic	*P*	FDR
		18-34yr	35-44yr	45-55yr	>55yr			
		(*n* = 986)	(*n* = 675)	(*n* = 330)	(*n* = 125)			
Microbial density, *n* (%)						χ^2^ = 50.112	<0.001	<0.001
Normal	1104 (70.860)	559 (76.054)	369 (73.360)	134 (54.918)	42 (55.263)			
Abnormal	454 (29.140)	176 (23.946)	134 (26.640)	110 (45.082)	34 (44.737)			
Microbial diversity, *n* (%)						χ^2^ = 75.961	<0.001	<0.001
Normal	1433 (91.977)	696 (94.694)	473 (94.036)	212 (86.885)	52 (68.421)			
Abnormal	125 (8.023)	39 (5.306)	30 (5.964)	32 (13.115)	24 (31.579)			
Dominant bacteria, *n* (%)						NA*	<0.001	0.001
Gram-Positive Rods	835 (53.594)	436 (59.320)	300 (59.642)	82 (33.607)	17 (22.368)			
Gram-Negative Rods	565 (36.264)	244 (33.197)	171 (33.996)	123 (50.410)	27 (35.526)			
Gram-Positive Cocci	36 (2.311)	15 (2.041)	12 (2.386)	3 (1.230)	6 (7.895)			
Others	51 (3.273)	25 (3.401)	12 (2.386)	9 (3.689)	5 (6.579)			
Not Found	71 (4.557)	15 (2.041)	8 (1.590)	27 (11.066)	21 (27.632)			
BV, *n* (%)						χ^2^ = 24.429	<0.001	<0.001
–	990 (63.543)	488 (66.395)	332 (66.004)	121 (49.590)	49 (64.474)			
+	568 (36.457)	247 (33.605)	171 (33.996)	123 (50.410)	27 (35.526)			
AV, *n* (%)						NA*	0.008	0.009
–	1463 (93.902)	692 (94.150)	479 (95.229)	228 (93.443)	64 (84.211)			
+	95 (6.098)	43 (5.850)	24 (4.771)	16 (6.557)	12 (15.789)			
VVC, *n* (%)						χ^2^ = 34.348	<0.001	<0.001
–	1314 (84.339)	589 (80.136)	424 (84.294)	226 (92.623)	75 (98.684)			
+	244 (15.661)	146 (19.864)	79 (15.706)	18 (7.377)	1 (1.316)			
TV, *n* (%)						NA*	0.001	0.002
–	1546 (99.230)	733 (99.728)	501 (99.602)	236 (96.721)	76 (100.000)			
+	12 (0.770)	2 (0.272)	2 (0.398)	8 (3.279)	0 (0.000)			
CV, *n* (%)						NA*	0.567	0.567
–	1535 (98.524)	721 (98.095)	496 (98.608)	242 (99.180)	76 (100.000)			
+	23 (1.476)	14 (1.905)	7 (1.392)	2 (0.820)	0 (0.000)			
pH, n (%)						χ^2^ = 70.438	<0.001	<0.001
<4.6	657 (42.169)	328 (44.626)	254 (50.497)	68 (27.869)	7 (9.211)			
≥4.6	901 (57.831)	407 (55.374)	249 (49.503)	176 (72.131)	69 (90.789)			
H_2_0_2_, n (%)						χ^2^ = 16.189	0.001	0.002
–	302 (19.384)	139 (18.912)	118 (23.459)	41 (16.803)	4 (5.263)			
+	1256 (80.616)	596 (81.088)	385 (76.541)	203 (83.197)	72 (94.737)			
Leucocyte esterase, n (%)						χ^2^ = 12.676	0.005	0.006
–	213 (13.671)	118 (16.054)	68 (13.519)	24 (9.836)	3 (3.947)			
+	1345 (86.329)	617 (83.946)	435 (86.481)	220 (90.164)	73 (96.053)			
Sialidase, n (%)						χ^2^ = 25.484	<0.001	<0.001
–	1220 (78.306)	587 (79.864)	406 (80.716)	162 (66.393)	65 (85.526)			
+	338 (21.694)	148 (20.136)	97 (19.284)	82 (33.607)	11 (14.474)			

*Fisher’s exact test was applied when expected cell frequencies <5 or total sample size <40. All P-values were corrected for multiple comparisons using the Benjamini-Hochberg false discovery rate (FDR) method. q<0.05 was considered statistically significant.

BV, Bacterial Vaginosis; AV, Aerobic Vaginitis; VVC, Vulvovaginal Candidiasis; TV, Trichomonas Vaginalis; CV, Cytolytic Vaginosis.

### 3.4 Age-stratified microecological risk factors of HPV acquisition

To identify independent risk factors of HPV infection across different life stages, we performed univariable and multivariable logistic regression analyses in the HPV-screened subset (n = 2,116). Univariable analysis (**Table 5**, **Supplementary Figure 3a**) revealed that older age (>55 years vs. 18–34 years: OR = 1.888, 95% CI: 1.277–2.771, P<0.05), BV (OR = 1.240, 95% CI: 1.018–1.511, P<0.05), VVC (OR = 0.637, 95% CI: 0.467–0.856, P<0.05), H_2_O_2_ positivity (OR = 0.768, 95% CI: 0.611–0.971, P<0.05), and sialidase positivity (OR = 1.413, 95% CI: 1.132–1.759, P<0.05) were significantly associated with HPV status.

**Table 5:** Univariate logistic regression analysis.

Variable	Estimate	*S.E*	OR(95%*CI*)	Z	*P*
AgeGroup					
18-34	Ref				
35-44	0.001	0.115	1.001 (0.799, 1.253)	0.011	0.991
45-55	0.032	0.145	1.032 (0.774, 1.368)	0.218	0.828
>55	0.635	0.197	1.888 (1.277, 2.771)	3.222	0.001
BV					
-	Ref				
+	0.215	0.101	1.240 (1.018, 1.511)	2.138	0.033
AV					
-	Ref				
+	-0.459	0.242	0.632 (0.384, 0.996)	-1.896	0.058
VVC					
-	Ref				
+	-0.451	0.154	0.637 (0.467, 0.856)	-2.925	0.003
TV					
-	Ref				
+	0.493	0.478	1.637 (0.606, 4.090)	1.03	0.303
CV					
-	Ref				
+	0.294	0.37	1.342 (0.626, 2.710)	0.795	0.426
pH					
-	Ref				
+	0.121	0.101	1.129 (0.927, 1.376)	1.202	0.229
H_2_O_2_					
-	Ref				
+	-0.264	0.118	0.768 (0.611, 0.971)	-2.229	0.026
Leucocyte esterase					
-	Ref				
+	-0.026	0.143	0.975 (0.740, 1.295)	-0.181	0.857
Sialidase					
-	Ref				
+	0.346	0.112	1.413 (1.132, 1.759)	3.076	0.002
Microbial density					
Normal	Ref				
Abnormal	0.368	0.104	1.445 (1.178, 1.770)	3.547	<0.001
Microbial diversity					
Normal	Ref				
Abnormal	0.142	0.174	1.153 (0.814, 1.612)	0.819	0.413

BV, Bacterial Vaginosis; AV, Aerobic Vaginitis; VVC, Vulvovaginal Candidiasis; TV, Trichomonas Vaginalis; CV, Cytolytic Vaginosis.

While univariate analyses identified multiple microecological parameters associated with HPV status, we next sought to determine independent risk factors using a robust multivariate analytical framework. LASSO regression with 10-fold cross-validation (λ = 0.007) was applied to select the most informative variables from the complete set of microecological parameters, addressing potential overfitting and multicollinearity (**Supplementary Figures 3b, c**; **Supplementary Table S6**). By combining clinical diagnostic evaluation with the LASSO selection process, we identified six significant microecological factors associated with HPV status: VVC, AV, BV, H_2_O_2_, TV, and Age Group.

**Figure 2:**
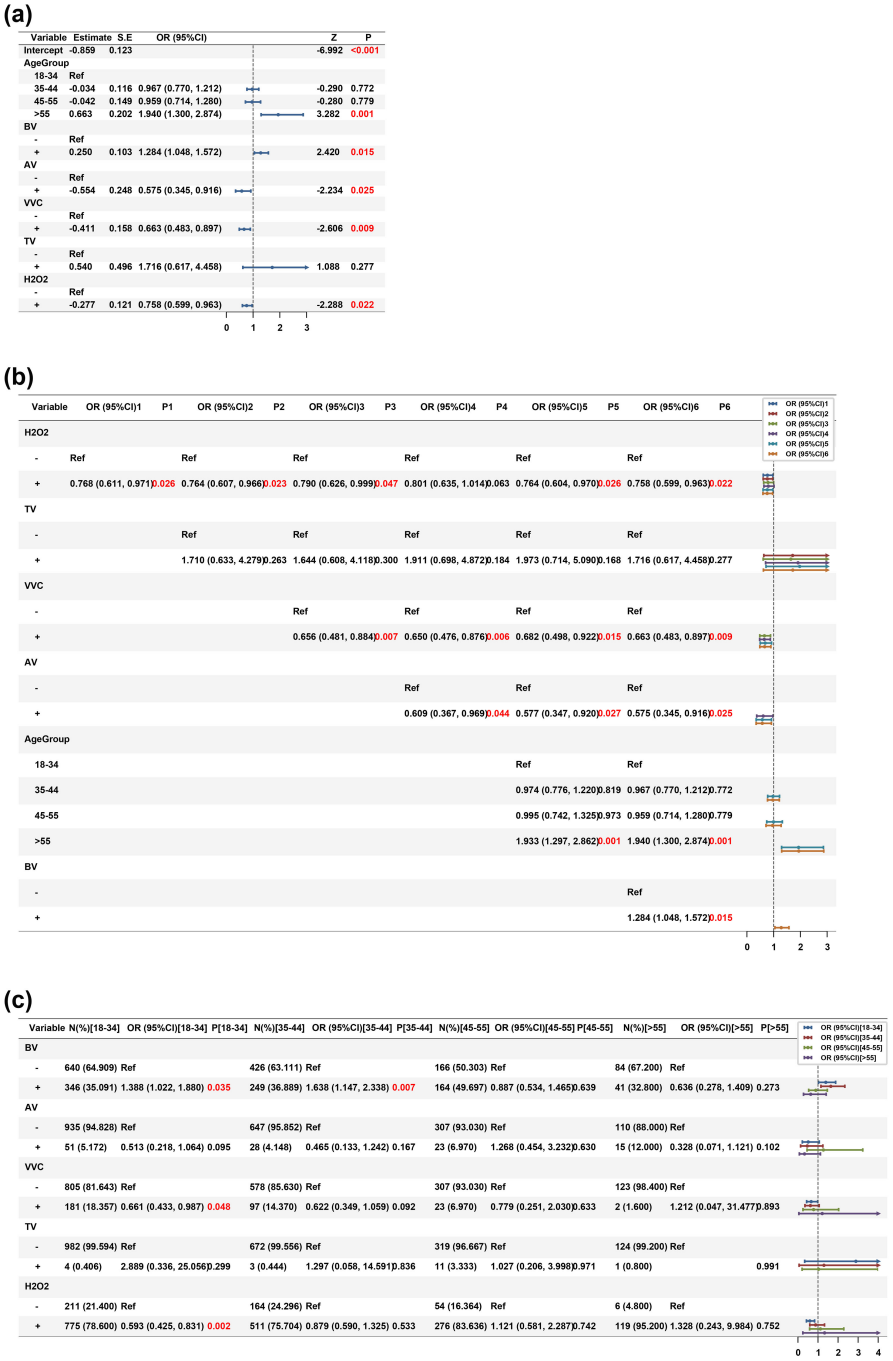
Multivariable analysis and age-stratified associations of vaginal factors with HPV infection risk. Forest plots depicting odds ratios (OR) with 95% confidence intervals (95% CI) for vaginal microecological parameters associated with HPV infection. **(a)** Multivariate logistic regression model incorporating variables retained by LASSO selection (age group, BV, AV, VVC, TV, H_2_O_2_). The x-axis represents OR on a log_2_ scale; values >1 indicate increased HPV risk. Statistically significant associations (q<0.05) are highlighted. **(b)** Stepwise variable adjustment analysis showing progressive model refinement. **(c)** Age-stratified subgroup analyses reveal distinct risk factor profiles across reproductive life stages. Sample sizes: n=986 (18–34 years), n=675 (35–44 years), n=330 (45–55 years), n=125 (>55 years). All analyses controlled for multiple comparisons using Benjamini-Hochberg FDR correction. BV, Bacterial vaginosis; AV, Aerobic vaginitis; VVC, Vulvovaginal candidiasis; TV, Trichomoniasis; H_2_O_2_, Hydrogen Peroxide; OR, Odds ratio; CI, Confidence interval; LASSO, Least Absolute Shrinkage and Selection Operator.

Multivariate logistic regression analysis, incorporating all retained variables (**Figure 2a**, **Supplementary Table S7**), revealed that after mutual adjustment, BV remained a significant independent risk factor for HPV infection (aOR = 1.284, 95% CI: 1.048–1.572, P<0.05). Conversely, both AV (aOR = 0.575, 95% CI: 0.345–0.916, P<0.05) and VVC (aOR = 0.663, 95% CI: 0.483–0.897, P<0.05) demonstrated independent protective effects against HPV infection. Age emerged as a critical modifier, with women >55 years showing a 1.940-fold higher odds of HPV positivity compared to the 18–34 years reference group (95% CI: 1.300–2.874, P<0.05). Additionally, H_2_O_2_ positivity was independently associated with reduced HPV risk (aOR = 0.758, 95% CI: 0.599–0.963, P<0.05). All VIF were <10, indicating no substantial multicollinearity.

To assess the stability of these associations, we performed stepwise variable adjustment analysis (**Figure 2b**). The protective effect of VVC persisted throughout all adjustment stages, with risk reduction ranging from 32.3% to 34.4% as additional variables were sequentially included. Similarly, AV’s protective association remained stable (with a 39.1-42.5% risk reduction) after adjusting for VVC and TV. The independent risk effect of BV became apparent only after controlling for AV and VVC (model 6: aOR = 1.284, P<0.05), suggesting that confounding effects were present in the unadjusted analyses. These findings demonstrate that associations between vaginal microecological states and HPV infection are complex and context-dependent.

### 3.5 Age-stratified risk factor profiles reveal distinct windows of susceptibility

Recognizing that HPV-microbiota interactions are age-dependent, we performed subgroup logistic regression analyses stratified by age group (**Figure 2c**). This analysis revealed striking heterogeneity in risk factor profiles across the female lifespan.

In women aged 18–34 years (n=986), BV remained the dominant independent risk factor (aOR = 1.388, 95% CI: 1.022–1.880, P<0.05), while VVC showed a strong protective effect (aOR = 0.661, 95% CI: 0.433–0.987, P<0.05). Notably, H_2_O_2_ positivity was also protective in this age group (aOR = 0.593, 95% CI: 0.425–0.831, P<0.05). This suggests that in younger women, maintaining H_2_O_2_-producing *Lactobacilli* and controlling anaerobic overgrowth are critical for HPV prevention.

The 35–44 years age group (n=675) exhibited the most pronounced risk pattern, with BV demonstrating significantly elevated odds of HPV infection (aOR=1.638, 95% CI: 1.147–2.338, P<0.05). This age window corresponds to the perimenopausal transition, where estrogen fluctuations may destabilize the dominant microbiota, creating a permissive environment for HPV persistence. Notably, the magnitude of BV’s effect was 18.0% higher than in younger women and reached statistical significance despite a smaller subgroup sample size. VVC showed a similar protective direction but was marginally non-significant (aOR = 0.622, 95% CI: 0.349–1.059, P = 0.092).

In stark contrast, women aged 45–55 years (n=330) and >55 years (n=125) showed no significant independent risk factors (all P>0.05), indicating that age-related hormonal changes and universal dysbiosis may overwhelm infection-specific risk signals. These age-stratified models underscore that HPV prevention strategies must be tailored to specific life stages: younger women benefit from VVC control and *Lactobacilli* support, while midlife women require intensified BV surveillance and targeted anaerobic suppression.

### 3.6 Age-specific microbiota dysbiosis and functional reprogramming revealed by targeted 16S rRNA sequencing

To functionally validate the age-specific HPV-microbiota associations identified in our large-scale clinical analysis (n = 23,672), we performed targeted 16S rRNA sequencing on a validation cohort (n = 88 women, stratified into 8 groups by age and HPV status, n = 11 per group). *Post-hoc* power analysis confirmed that this sample size provides 95.3% statistical power (Cohen’s d = 1.62, α = 0.05) to detect the primary hypothesis of altered Shannon diversity in the midlife group (35–44 years), exceeding the 80% threshold recommended for validation studies. However, we transparently acknowledge that this cohort is underpowered for detecting subtle shifts in low-abundance taxa (<5% abundance) and for robust Community State Type (CST) classification, which would require n>200. Therefore, we focused on community-level patterns and pre-specified metabolic pathways where effect sizes were large and biologically plausible.

**Figure 3:**
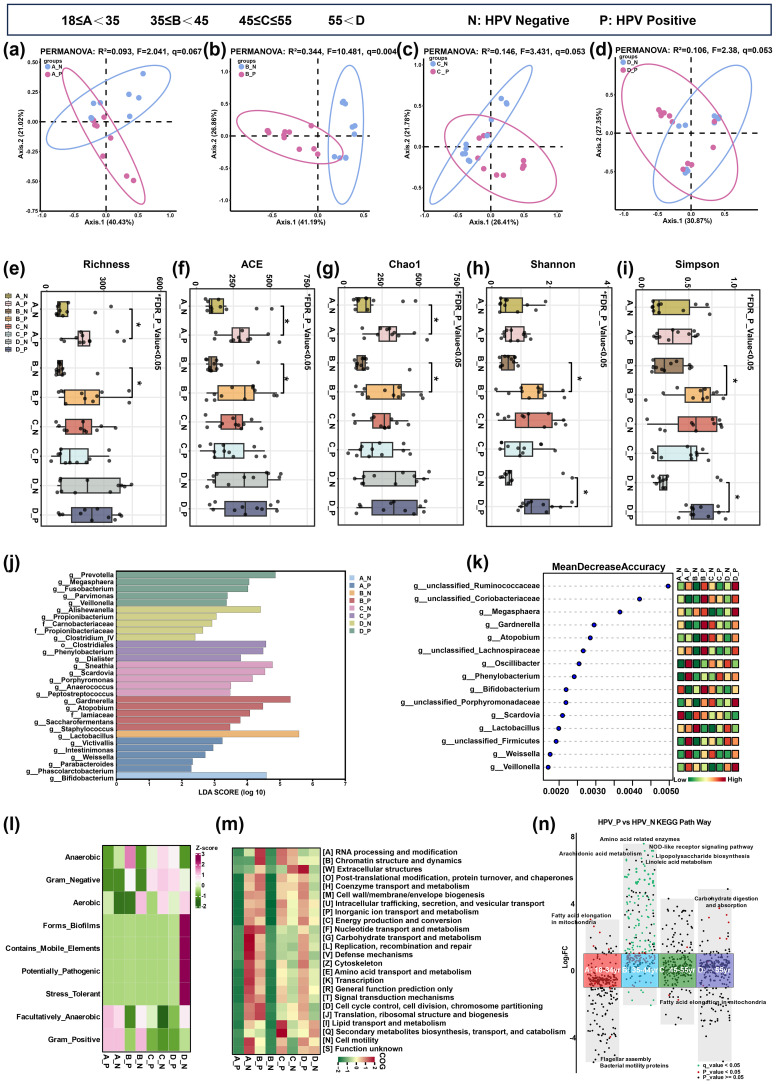
Age-gated microbiome dysbiosis and functional reprogramming in HPV-infected women. 16S rRNA sequencing analysis of vaginal microbiota across four age strata. **(a–d)** Principal Coordinates Analysis (PCoA) based on weighted UniFrac distances for age groups 18-34 **(A)**, 35-44 **(B)**, 45-55 **(C)**, and >55 **(D)** years; PERMANOVA statistics shown. **(e–i)** α-diversity indices (Richness, ACE, Chao1, Shannon, Simpson) comparing HPV-positive (P) vs. HPV-negative (N) groups (*q<0.05, **q<0.01, ***q<0.001). **(j)** LEfSe analysis (LDA score > 3.5) revealed enrichment of *Gardnerella* and *Prevotella* in HPV-positive groups versus *Lactobacillus* in HPV-negative groups. **(k)** Random Forest mean decrease accuracy for taxa discriminating HPV status. **(l)** BugBase phenotypic predictions of anaerobic and pathogenic potentials. **(m)** COG functional categories inferred by PICRUSt2. **(n)** KEGG pathway heatmap showing log_2_ fold-changes in HPV-positive vs. HPV-negative women; only FDR-significant pathways (q<0.05) in the 35–44 years group are highlighted. HPV, Human papillomavirus; PCoA, Principal Coordinates Analysis; LEfSe, Linear Discriminant Analysis Effect Size; LDA, Linear Discriminant Analysis; KEGG, Kyoto Encyclopedia of Genes and Genomes.

PCoA based on weighted UniFrac distances revealed age-specific structural segregation (**Figures 3a–d**). HPV status did not significantly separate microbiota composition in the youngest group (18–34 years, PERMANOVA: R²=0.093, F = 2.041, q=0.067), but showed clear separation in older groups with effect sizes increasing with age (35–44 years: R²=0.344, F = 10.481, q=0.004; 45–55 years: R²=0.146, F = 3.341, q=0.053; >55 years: R²=0.106, F = 2.38, q=0.053). This age-gated HPV effect aligns with our epidemiological findings and supports the existence of a critical midlife vulnerability window.

Alpha diversity metrics demonstrated heterogeneous and age-specific patterns (**Figures 3e–i**). FDR-corrected analysis confirms that HPV-associated microbial diversity elevation was primarily concentrated in midlife and postmenopausal women, reinforcing the critical 35–44 years window where viral and hormonal insults synergistically drive dysbiosis. Younger women maintain diversity homeostasis regardless of HPV status, likely due to estrogen-protected *Lactobacillus* dominance.

Taxa identified by Random Forest (Mean Decrease Accuracy >0.5) were cross-validated by LEfSe (LDA >3.0), which identified enrichment of anaerobic taxa (e.g., *Gardnerella, Atopobium*) in HPV-positive women aged 35–44 years, while *Lactobacillus* dominated HPV-negative groups (**Figures 3j, k**, q<0.05). BugBase phenotypic prediction revealed higher abundances of anaerobic and pathogenic phenotypes in aged 35–44 years HPV-positive women, contrasting with *Lactobacilli*-dominated phenotypes in HPV-negative women (**Figure 3l**, q<0.05).

PICRUSt2-based COG functional analysis (**Figure 3m**, q<0.05) showed that HPV-positive women had enhanced signal transduction and carbohydrate metabolism pathways, while defense mechanisms were enriched in HPV-negative women. NSTI scores for all 88 samples ranged from 0.003 to 0.116 (mean 0.089, **Supplementary Table S4**), indicating good prediction accuracy. These findings suggest an age-dependent, HPV-associated dysregulation of the vaginal microbiota composition, characterized by anaerobic expansion, altered metabolic activity, and reduced defense capacity.

Comprehensive KEGG pathway analysis (**Figure 3n**) demonstrated that HPV-induced microbial metabolic alterations exhibited striking age dependency. In reproductive-aged women (18–34 years, Group A), HPV infection showed nominal upregulation of fatty acid elongation (FC = 2.75, P = 0.048), although no pathways survived FDR correction. The most profound metabolic dysregulation emerged in mid-adulthood (35–44 years, Group B), where HPV-positive women exhibited significant enrichment of pro-inflammatory pathways including NOD-like receptor signaling (FC = 62.06, q < 0.001), arachidonic acid metabolism (FC = 33.27, q = 0.038), and lipopolysaccharide biosynthesis (FC = 37.30, q = 0.028). Concurrent upregulation of amino acid metabolism and downregulation of ethylbenzene degradation indicated disrupted detoxification capacity. Strikingly, the apoptosis pathways were suppressed (FC = 0.35, q = 0.046), suggesting impaired clearance of infected cells. Peri-menopausal women (45–55 years, Group C) and postmenopausal women (>55 years, Group D) displayed minimal HPV-associated metabolic alterations, with fatty acid elongation showing nominal decrease in Group C (FC = 0.30, P = 0.036) and carbohydrate digestion and absorption (FC = 13.127, P = 0.01) in Group D but with no FDR-significant pathways in both group. This metabolic perturbation pattern, characterized by amplified inflammatory lipid signaling, compromised cellular defense, and enhanced microbial virulence, specifically implicates mid-adulthood as a critical window for HPV-driven metabolic reprogramming of the vaginal microenvironment.

### 3.7 Integrated microecological and metabolic dynamics underlie age-stratified HPV-host interactions

Vaginal microecological parameters exhibited systematic age-dependent restructuring, characterized by a progressive decline in microbial density (normal flora prevalence: 78.7% in 18-34years vs. 48.8% in >55years, χ²=256.2, q<0.001) and increasing pH alkalinization (pH≥4.6: 73.5% to 90.0%, χ²=134.7, q<0.001). Crucially, HPV infection was associated with distinct functional alterations that aligned with specific microecological perturbations, with maximal convergence in women aged 35–44 years. FDR-corrected pathway analysis revealed that in 35–44 years group (**Supplementary Table S8**), elevated BV prevalence (HPV+ vs. HPV-: 45.35% vs. 33.99%, χ² = 7.096, q=0.032) directly corresponded to 37.3-fold upregulation of lipopolysaccharide biosynthesis (FDR = 0.028), driving endotoxin-mediated inflammation. Concurrently, enhanced sialidase activity (HPV+ vs HPV-: 31.4% vs 19.28%, χ² = 10.826, q = 0.012) synergized with metabolic reprogramming, including a 33.3-fold activation of arachidonic acid metabolism (q = 0.038) and a 62.1-fold induction of NOD-like receptor signaling (q < 0.001), collectively generating a pro-inflammatory milieu. Defense mechanisms were compromised, as evidenced by the suppression of apoptosis (FC = 0.35, FDR = 0.046), which mirrored the deficiency in H_2_O_2_ production. Conversely, downregulated ethylbenzene degradation (FC = 0.41, FDR = 0.002) indicated impaired xenobiotic detoxification. Notably, only the midlife group (35-44years) exhibited FDR-significant metabolic pathways, while younger and older groups showed nominal or non-significant changes, reinforcing the 35–44 years critical vulnerability window.

Younger women (18–34 years) showed merely a slight, non-significant increase in fatty acid elongation (FC = 2.75, P = 0.048), aligning with isolated H_2_O_2_ deficiency but lacking coordinated metabolic dysregulation. The absence of FDR-significant pathways in this age group reflects robust estrogen-mediated resilience, where lactobacilli-dominant communities buffer against viral insults. Peri-menopausal (45–55 years) and post-menopausal (>55 years) groups showed attenuated interactions, consistent with dominant age-related microecological reorganization overriding virus-specific effects. This integrated analysis established a mechanistic triad: anaerobic dysbiosis (BV-associated Gram-negative rods); endotoxin-driven metabolic inflammation (LPS/AA pathways); immunosuppressive microenvironment (apoptosis inhibition), and peaking during the estrogen-fluctuating 35–44 years period to facilitate HPV persistence.

## 4 Discussion

Our large-scale, age-stratified analysis (n = 23,672) provides resolution of vaginal microecology across the reproductive lifespan, revealing that HPV infection functions as an active modulator of age-related microbial transitions, rather than merely a passive correlate. We identify a critical midlife vulnerability window (35–44 years) where viral and hormonal insults synergistically accelerate dysbiosis, extending beyond descriptive aging models to offer a mechanistic foundation for precision gynecologic interventions.

Our observation that H_2_O_2_ loss is the strongest youth risk factor aligns mechanistically with Chee et al. ‘s finding and provides age-specific translational evidence (Chee et al., 2020). The linear decline in *Lactobacillus* dominance (78.7% to 48.8%) and progressive pH alkalinization corroborate CST transitions described by Brotman et al (Brotman et al., 2018), where postmenopausal women predominantly exhibit *Lactobacillus*-depleted CST IV-A, and mechanistically align with estrogen-dependent glycogen depletion and *Lactobacillus* loss detailed by Muhleisen and Herbst-Kralovetz (Muhleisen and Herbst-Kralovetz, 2016; Di Paola et al., 2017). However, our cohort uniquely captures a novel midlife peak in microbial diversity (45–55 years) preceding postmenopausal stabilization, likely reflecting perimenopausal hormonal volatility. This non-linear trajectory challenges linear aging models and suggests perimenopause constitutes a distinct ecological state requiring denser longitudinal sampling to resolve temporal instability versus true community complexity.

Critically, HPV infection actively remodels these age-related dynamics. While Gajer et al (Gajer et al., 2012). characterized the temporal dynamics in healthy, virus-negative women, our multivariate analysis demonstrates that HPV exacerbates the “estrogen cliff” effect, specifically in midlife (35–44 years), synergistically accelerating BV phenotypes and sialidase activity, independent of chronological age. This viral-bacterial synergy appears to be mediated by immune-metabolic reprogramming: PICRUSt2 functional predictions reveal that HPV-positive midlife microbiomes exhibit a 37.3-fold upregulation of LPS biosynthesis pathways and a 62.1-fold activation of NOD-like receptor signaling, creating a pro-inflammatory feedforward loop that compromises barriers and innate immunity. However, we caution that these predictions, although hypothesis-generating with acceptable NSTI scores (0.003–0.116), represent metabolic potential requiring metagenomic validation. The apparent attenuation of dysbiosis severity in HPV-positive elderly women is intriguing but statistically limited by the small sample size, precluding robust multivariate adjustment according to Vittinghoff et al.’s “10:1 rule.” (Vittinghoff and McCulloch, 2007).

The mechanisms underlying this HPV-microbiota-age interaction appear multifactorial. A study reported that several non-*Lactobacillus* species can serve as biomarkers for HPV infection, indicating a complicated interaction between HPV and vaginal microbiota (Cheng et al., 2020). Our observation that VVC prevalence inversely correlates with HPV infection (10.57% vs. 15.66%, aOR = 0.641) aligns with Feng et al (Feng et al., 2023). report of VVC’s protective effect against non-16/18 HPV subtypes (OR = 0.562, 95% CI: 0.380–0.831). We hypothesize that this may reflect either immune-mediated competition, where a *Candida*-elicited Th17 response enhances mucosal antiviral defense, or direct ecological competition for niche space, though the cross-sectional nature of our study precludes definitive causal inference.

BV, the most prevalent dysbiosis (293/23,672, 1.24%), is characterized by a loss of *Lactobacillus* and proliferation of anaerobes. Bacteria frequently associated with BV produce sialidases and mucinases that damage genital epithelia and disrupt innate immunity, compromising barrier function. Our data showed dysbiosis incidence increased with age, with perimenopausal and postmenopausal women showing significantly higher BV/AV rates than premenopausal women, consistent with Maswanganye et al (Maswanganye et al., 2024). and Liu et al.’s (Liu et al., 2023) meta-analyses reporting statistical BV-HPV associations. However, our multivariate model revealed that AV and VVC presence confound this relationship, with BV aOR = 1.284, demonstrating complex confounding directions. This aligns with Monica et al.’s consideration of CST IV-BV as an HPV persistence risk factor and identification of the *Gardnerella* sialidase gene as a microbial persistence marker (Cheng et al., 2020).

Our microscopy-based morphotype-dominance framework, rather than CST assignment, was driven by clinical translatability. All 23,672 samples were derived from routine testing, where microscopy remains the only universally accessible low-resource tool. While Ravel’s (Ravel et al., 2011) CST taxonomy provides research-grade resolution, our 16S subcohort (n=88) was powered to detect dominant taxa (>5% abundance). Still, it lacked statistical power (<20%) for low-prevalence CST subtypes, such as IV-B, making robust classification inappropriate. Future studies with larger sequencing cohorts (n>200) should integrate CST assignment to map age-dependent BV/sialidase associations to CST IV-B and determine whether HPV-driven H_2_O_2_-producing *Lactobacilli* suppression corresponds to CST I/III/IV transitions, bridging mechanistic insights with standardized taxonomy for precision screening.

Our bimodal HPV infection pattern, peaking in young (<30 years) and elderly (≥60 years) women, aligns with established demographics and Li et al.’s (Li et al., 2024) Chongqing cohort, reinforcing age-dependent susceptibility as a universal Chinese population feature. Regarding confounding, Brotman et al (Brotman et al., 2018). reported increased CST IV-A postmenopause, consistent with our >55 years *Lactobacillus* proportion (48.8%), yet our 45–55 years diversity elevation contradicts linear models, likely reflecting short-term perimenopausal hormonal effects. This subgroup’s limited size (n = 330) necessitates larger validation (Doerflinger et al., 2014; Borgdorff et al., 2016; Soper, 2020).

Critical limitations necessitate transparent acknowledgment. First, our cross-sectional design cannot establish temporal relationships; thus, all associations remain correlative, not causal. While LASSO-regularized regression controlled confounding, it cannot resolve the simultaneous measurement of exposure and outcome. Second, the exclusion of vaccinated participants eliminated confounding but precluded evaluation of vaccine-microbiota interactions, which is a critical modifier missing from current literature. Third, the 16S validation cohort, although adequately powered (95.3%) for our primary hypothesis, is underpowered for rare taxa and robust CST classification, limiting the generalizability of microbial signatures. Fourth, PICRUSt2 functional predictions, though hypothesis-generating, require metagenomic validation to confirm actual metabolic pathway activity. The low statistical power observed within the 18–34 years age group (5.6%) quantitatively reflects the lack of association found in our data for that demographic. However, we acknowledge that a larger sample size within this group could potentially reveal subtle metabolic shifts or dynamics involving low-abundance microbial species that precede the development of overt dysbiosis. Nevertheless, our large-scale epidemiological data provide robust effect size estimates that guide future targeted investigations.

Furthermore, while our differential abundance analysis employed Random Forest and LEfSe methodologies, selected for their stability in smaller validation cohorts, we recognize that alternative algorithms, such as ANCOM-BC2 and MaAsLin2, offer superior control over compositional data biases and confounding variables. The limited sample size available in this study (n = 11 per group) did not provide sufficient statistical power to implement these more conservative methods reliably. To address these limitations and advance our understanding, we are actively recruiting participants for an expanded prospective cohort focused specifically on young women. This initiative aims to explore fine-scale interactions between HPV and the vaginal microbiome and to identify potential early biomarkers of susceptibility. The relationship between vaginal infections and persistent oncogenic HPV infection requires further characterization.

Future research should establish causality through longitudinal cohorts with pre-HPV infection baseline microbiota sampling, allowing for time-to-event analysis of HPV acquisition and clearance. Mechanistic insights should be pursued using germ-free and humanized mouse models colonized with clinical HPV-positive microbiomes to test whether specific anaerobic bacteria facilitate viral persistence, alongside *in vitro* or *ex vivo* studies that quantify the effects of HPV viral proteins (E6/E7) on *Lactobacillus* and anaerobic bacterial metabolism, as well as epithelial-immune crosstalk. Randomized controlled trials evaluating the efficacy of CST-guided *Lactobacillus* probiotics in midlife HPV-positive women are also warranted. Expanding sequencing efforts in larger cohorts (n>200 per group) will allow the application of ANCOM-BC2 for robust validation of taxonomic findings and MaAsLin2 to effectively model covariates like age and HPV status as mixed effects, ensuring the reliable identification of microbial biomarkers. Our follow-up study will further explore vaginal microecology-HPV relationships to inform treatment and cervical lesion prevention, providing personalized strategies for HPV management across the female lifespan.

Despite these limitations, our study delineates age-specific clinical implications. Midlife women (35–55 years) emerge as a priority group for enhanced surveillance of anaerobic dysbiosis and BV biomarkers. Interventions that restore *Lactobacillus* dominance or suppress anaerobes may be particularly effective during this estrogen-fluctuating window. For younger women, maintaining a healthy microbiota to prevent VVC may indirectly support defenses against HPV. In contrast, elderly women may require combined strategies that focus on microbiota restoration and mucosal health. Ultimately, this work frames the vaginal microenvironment not as a static background but as a dynamic, age-stratified ecosystem where host hormonal status, resident microbiota, and viral pathogens engage in complex interactions that define periods of heightened risk and opportunity for targeted intervention.

## 5 Conclusion

In conclusion, our age-stratified analysis reveals that HPV infection actively modifies, rather than merely accompanies, the trajectory of vaginal microbiota aging, with a critical synergistic effect observed during the midlife window (35–44 years), where viral presence exacerbates the estrogen imbalance-driven decline in *Lactobacillus* dominance and accelerates dysbiotic states, such as bacterial vaginosis. These findings advocate for life-stage-specific clinical management, prioritizing dysbiosis surveillance and restoration in midlife, maintaining eubiosis in youth, and combining mucosal and microbial interventions in elderly women, while framing the vaginal microenvironment as a dynamic participant in HPV susceptibility. Future longitudinal and multi-omics studies are essential to validate the predicted functional pathways, establish causality, and integrate factors like vaccination status, ultimately paving the way for personalized, microbiota-informed strategies to mitigate HPV persistence and its clinical sequelae.

## Data Availability

The original contributions presented in the study are publicly available. This data can be found here: NCBI SRA under BioProject PRJNA1377879, with BioSample accession numbers SAMN53807656–SAMN53807743).
