# Supplementary material for: Age-specific vaginal microecological dysbiosis associated with HPV infection: a large-scale cross-sectional study with targeted functional sequencing validation
Source: Front Cell Infect Microbiol. 2026 Jan 21;15:1722367. doi: 10.3389/fcimb.2025.1722367 (PMC12868268; doi:10.3389/fcimb.2025.1722367)
Supplement: Supplementary file 1 [file DataSheet1.pdf]

Supplement Figure

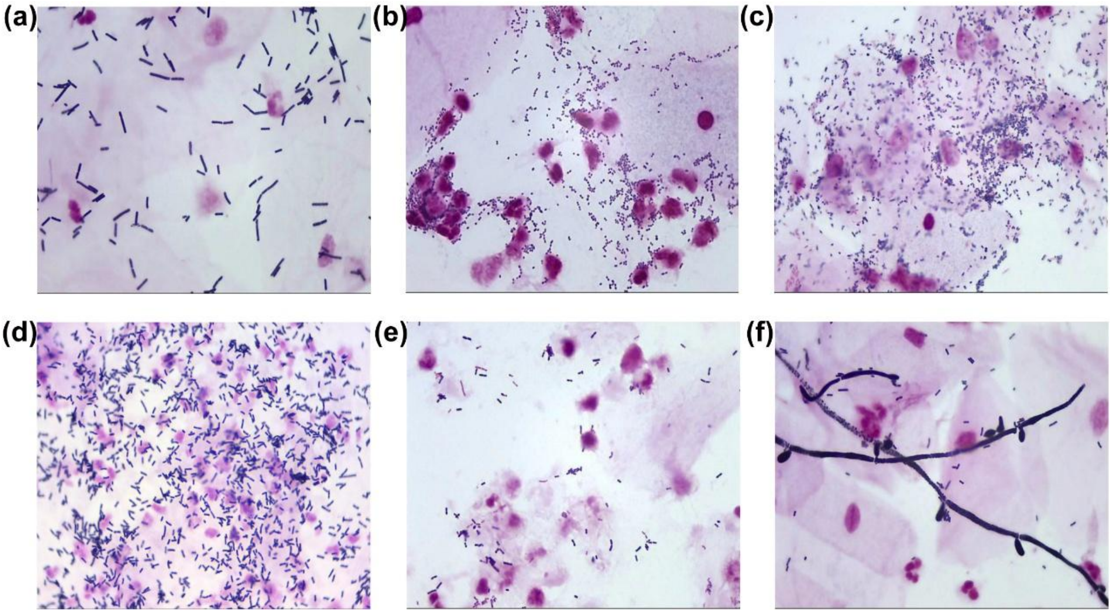

Supplementary Fig 1. Gram staining colposcopy of vaginal srecretion smear

(a) The microscopic image of patient with normal vaginal microecology with abundant lactobacillus distribution. (b) Microscopic images of AV patient. (c) The microscopic image of a patient with BV. (d) Microscopic image of CV patient, mainly showing an overabundance of lactobacillus with a large number of dissolved squamous epithelial cells. (e) The microscopic image of a patient with TV, showing the trichomonas pathogen. (f) Microscopic image of a patient with VVC.

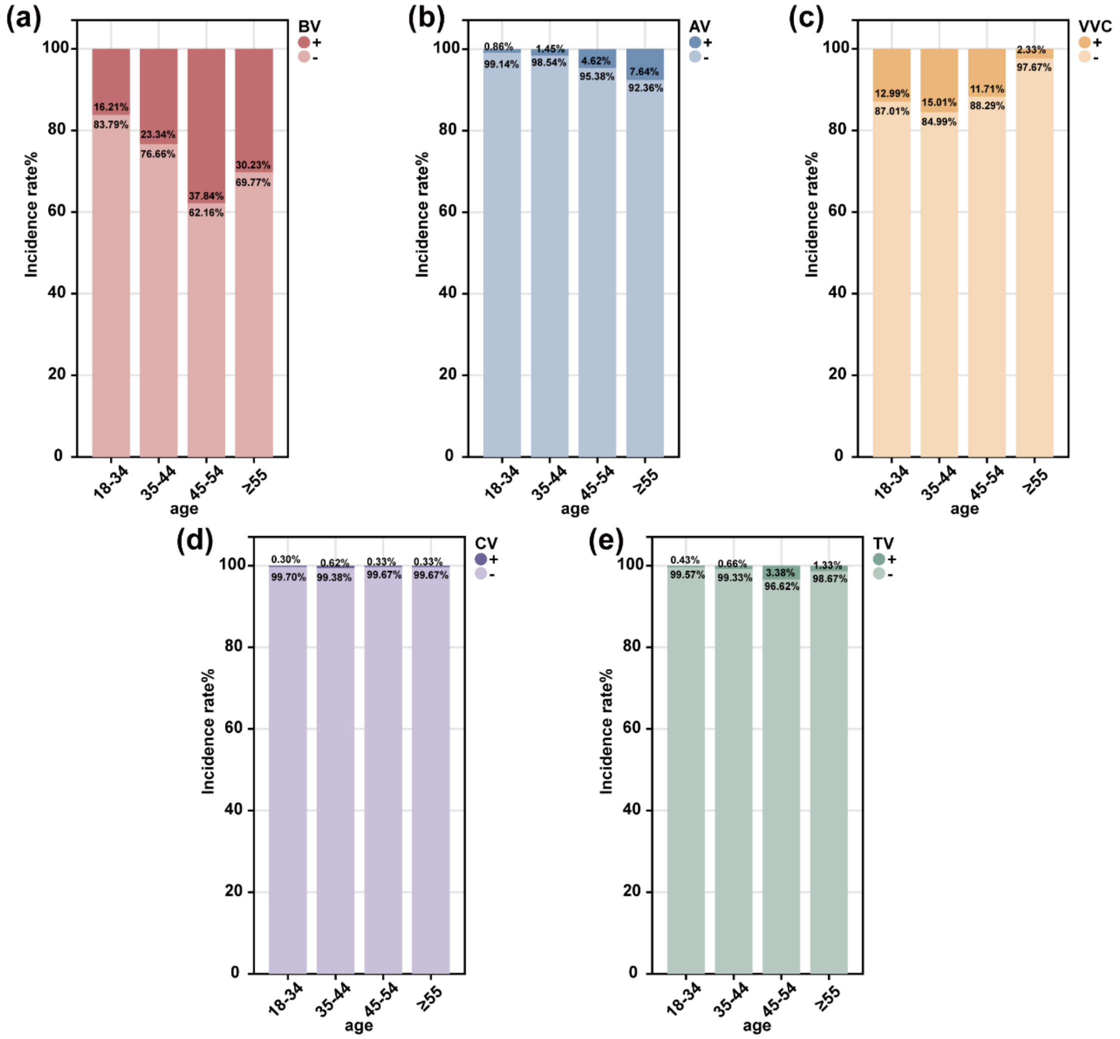

Supplementary Fig 2. The vaginal dysbiosis in women of different age.

(a) The infection state of BV in women of different ages. (b)The infection state of AV in women of different ages. (c) The infection state of VVC in women of different ages. (d)The infection state of CV in women of different ages. (e) The infection state of TV in women of different ages.

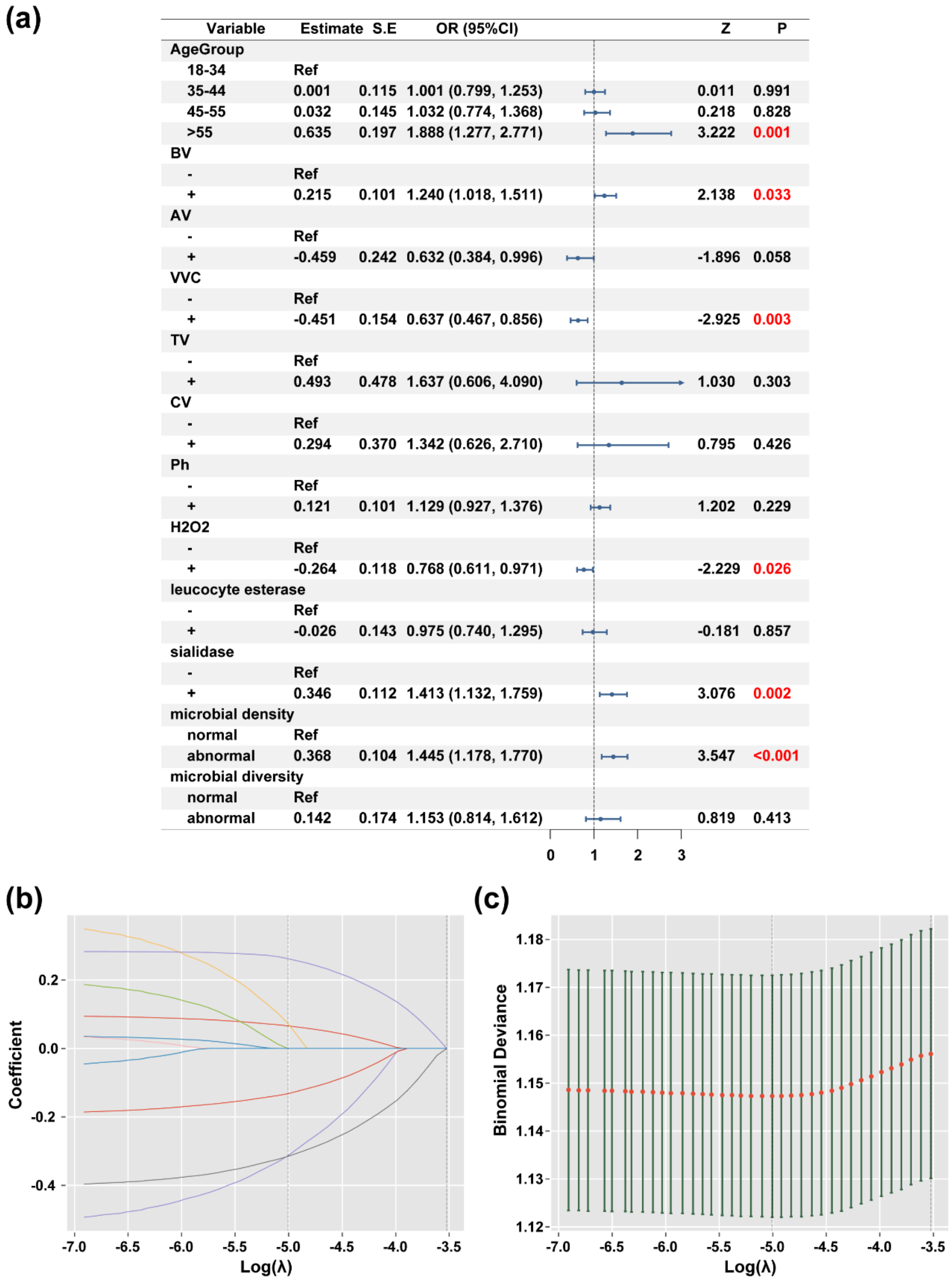

Supplementary Fig 3. Expanded Vaginal Microenvironment Analysis and LASSO Model Optimization for HPV Risk

(a) Univariate Logistic regression analysis of microecological parameters. (b) Ten-fold cross-validation plot illustrating mean squared error (MSE) as a function of log(λ). The optimal λ (0.007) was selected at minimum MSE. (c) Histogram of cross-validated MSE across λ values, demonstrating model stability. Variables with coefficients shrunk to zero were excluded from the final multivariate model.
